# Pepsin Egg White Hydrolysate Improves Glucose Metabolism Complications Related to Metabolic Syndrome in Zucker Fatty Rats

**DOI:** 10.3390/nu10040441

**Published:** 2018-04-03

**Authors:** Marta Garcés-Rimón, Cristina González, Gema Vera, José-A. Uranga, Rosina López-Fandiño, Visitación López-Miranda, Marta Miguel

**Affiliations:** 1 Instituto de Investigación en Ciencias de Alimentación (CIAL, CSIC-UAM), Madrid 28049, Spain; m.garces.rimon@gmail.com (M.G.-R.); rosina.lopez@csic.es (R.L.-F.); 2 Grupo de Investigación en Nutrición y Farmacología (URJC), Unidad Asociada al Instituto de Investigación en Ciencias de la Alimentación (CSIC), Madrid 28049, Spain; cristina.gonf@gmail.com (C.G.); gema.vera@urjc.es (G.V.); jose.uranga@urjc.es (J.-A.U.); visitacion.lopezmiranda@urjc.es (V.L.-M.); 3 Departamento de Ciencias Básicas de la Salud, Universidad Rey Juan Carlos, Alcorcón, Madrid 28922, Spain

**Keywords:** egg white hydrolysate, glucose homeostasis, insulin resistance, metabolic syndrome

## Abstract

The purpose of this study was to evaluate the effect of the administration of two egg white hydrolysates on glucose metabolism complications related to Metabolic Syndrome (MS) in Zucker fatty rats (ZFR). ZFR were given 750 mg/kg/day of egg white hydrolyzed with pepsin (HEW1) or with aminopeptidase (HEW2) for 12 weeks in their drinking water or just water. Zucker lean rats (ZLR), which received water, were used as a control. The presence of tactile allodynia, which is a sign of peripheral neuropathy, was assessed. Blood samples and pancreas were collected to determine the effect of the hydrolysates on glucose metabolism. The intake of HEW1 significantly lowered plasma insulin levels and improved the quantitative indexes of insulin resistance, insulin sensitivity, and pancreatic β-cell functionality (HOMA-IR, HOMA-β, and QUICKI, respectively), but non-significant changes were observed in group treated with HEW2. Compared to ZLR, ZFR showed tactile allodynia, but the consumption of both hydrolysates significantly increased mechanical sensitivity in ZFR. In conclusion, HEW1 pepsin could improve the glucose metabolism abnormalities associated with MS in obese Zucker rats.

## 1. Introduction

Metabolic syndrome (MS) is a common and complex disorder combining central obesity, insulin resistance, glucose intolerance, dyslipidemia, and hypertension. This pathology is considered a primary risk factor for diabetes and cardiovascular disease [1,2]. MS has become a major public health issue whose prevalence is increasing in Western countries particularly in developing areas undergoing rapid socio-environmental changes [2]. Obesity, and more specifically abdominal obesity, is pointed out as a primary contributor for acquiring insulin resistance, which causes hyperinsulinemia and may lead to the development of Diabetes Mellitus type 2 (DM2) [3].

The Zucker fatty rat (ZFR) is the most widely used animal model of obesity because, in addition to early overweight factors, it develops dyslipidemia, pre-hypertension, mild glucose intolerance, transient thermal hyperalgesia, and low-grade inflammation [4,5]. In fact, as well as resistance to the metabolic actions of insulin, these animals present hyperinsulinemia, which is detectable at three weeks old and persists throughout the animal’s life. These manifestations are similar to those found in human MS, which make this animal model a valuable tool for researching the pathophysiology as well as the pharmacological treatments of obesity-related glucose homeostasis. 

Various studies have highlighted the possibility of using food-derived compounds as natural products to control the metabolic complications related to MS including glucose homeostasis alterations [6]. In this context, bioactive peptides that are released during food processing or after digestion of food proteins from different sources can exert different biological activities [7]. Egg derived peptides have demonstrated angiotensin converting enzyme (ACE) inhibition, antioxidant and vasodilator properties [8,9,10,11], antihypertensive effects after short [12] and long term administration to spontaneously hypertensive rats [13], and beneficial properties on the lipid profile [14]. Recently, egg-derived peptides with α-glucosidase [15] or dipeptidyl peptidase IV (DPP-IV) inhibitory activities have also been described [16].

Our research group has obtained hydrolysates of egg white protein, which simultaneously possess several biological in vitro activities [17]. In particular, the hydrolysates of egg whites with pepsin or Peptidase 433P stood out for their ACE inhibition, antioxidant and bile acid-binding capacities, and both exhibited a moderate DDP IV-inhibitory activity. Additionally, in vivo studies related to the lipid metabolism were performed. In fact, egg white with pepsin reduced fat accumulation and hepatic steatosis and lowered plasmatic concentration of free fatty acids on ZFR [18], which suggests that they could target several disorders of a complex disease such as MS.

The aim of this study was to examine, from a multifunctional approach, the effect of these hydrolysates on glucose metabolism complications related to MS in ZFR.

## 2. Materials and Methods 

### 2.1. Preparation of Egg White Hydrolysates 

Pasteurized liquid hen egg white (Guillén, Spain) was treated with two different food grade enzymes, pepsin, and peptidase 433P to produce the hydrolysates named HEW1 and HEW2. To produce HEW1, egg white was acidified with a concentrated food grade HCl (37%) to pH 2.0 and treated with pepsin from the pork stomach (E.C. 3.4.23.1. BC Pepsin 1:3000 Biocatalysts, Cardiff, UK) at an enzyme to substrate ratio of 2:100 (*w*:*w*). After incubation at 37 °C under constant stirring in a thermostatic water bath for 8 h, pepsin was inactivated by increasing the pH to 7.0 with food grade NaOH (5N). To produce HEW2, egg white was adjusted to a pH of 7.0 with food grade NaOH (5N) and treated with aminopeptidase from Rhizopus oryzae (E.C. 3.4.11.1, Peptidase 433P, Biocatalysts, Cardiff, UK) at an enzyme to substrate ratio of 2:100 (*w*:*w*). Hydrolysis was carried out at 50 °C under constant stirring in a thermostatic water bath for 24 h and stopped by heating the samples at 95 °C for 15 min in a water bath, which was followed by cooling to room temperature.

Both hydrolysates were centrifuged for 15 min at 4500× *g* and the supernatants were stored at −20 °C until they were needed. The consistency of every hydrolysis process was checked by high performance liquid chromatography.

### 2.2. Animal Study

The experiments in the present study, which were designed to minimize the number of animals used and their suffering, were performed in strict accordance with the EC directive for the protection of animals used for scientific purposes (2010/63/UE) and the Spanish regulations (RD 53/2013), and they were approved by the Ethical Committee of Universidad Rey Juan Carlos de Madrid.

Thirty male eight-week-old Zucker fatty rats (ZFR) (250–275 g) and 10 male eight-week-old Zucker lean rats (ZLR) (150–175 g), purchased from the Charles River Laboratories (Charles River Laboratories, Barcelona, Spain) were used in this study. The animals were housed in standard transparent cages (60 cm × 40 cm × 20 cm) (2–3 animals/cage) furnished with wood shaving bedding, which was changed every 3–4 days. During the experimental period, until the rats were 20 weeks old, the animals were maintained under environmentally-controlled conditions (20 °C and 60% of humidity), with 12 h light/12 h dark cycles and they were fed ad libitum with a solid standard diet (Harlan Ibérica, Barcelona, Spain). ZLR received water until the 20th week of age while ZFR were randomly divided into 3 groups of 10 animals each, which received, respectively, water containing 750 mg/kg body weight/day of HEW1, 750 mg/kg body weight/day of HEW2, or solely water as a ZFR control. During the last week of treatment, the appearance of tactile allodynia (a sign of peripheral neuropathy) was assessed. At the end of the study, and after a fasting period of 12 h, the animals were sacrificed. Blood samples and the pancreas were collected to determine the effect of the hydrolysates on glucose metabolism.

### 2.3. Evaluation of Mechanical Sensitivity: Von Frey Hair Test

The development of peripheral neuropathy, which is usually associated with the impaired glucose intolerance characteristic of the pre-diabetic state, was assessed using the Von Frey hair test. The Von Frey hair test assesses mechanical sensitivity. Mechanical sensitivity was recorded at 19–20 weeks of age using an electronic Von Frey apparatus (EVF3, Bioseb, BP89, ChavilleCedez, France). Rats were placed individually on an elevated iron mesh in a clear plastic cage and allowed to adapt to the testing environment two days before assessment for at least 10 min per day. Von Frey hairs, small pieces of nylon rod, were applied to the plantar aspect of each hind paw below the mesh floor and the stimulus intensity that caused withdrawal responses was registered. The test was made 3 times per paw with an inter-stimulus interval of approximately 30 s. The mean of the 3 trials was used for data analysis. The person who performed the Von Frey hair test in the animals from the different groups did not know the treatment that had been administered to each of these groups. Mechanical allodynia was defined as a significant decrease in the Von Frey hairs withdrawal threshold evoked by mechanical stimuli. The apparatus had an upper cut-off limit for testing of 50 g.

### 2.4. Blood and Organ Collection

After a fasting period of 12 h, animals were sacrificed and blood and pancreas samples were obtained. Blood was collected into tubes containing lithium heparin as anticoagulant. These samples were centrifuged at 2500× *g* for 20 min at 4 °C to obtain plasma, which was divided into aliquots and kept frozen at −80 °C until analysis. Pancreas was processed to perform insulin secretory activity of isolated pancreatic islets and histopathological analysis.

### 2.5. Plasma Analytical Procedures: Glucose and Insulin Concentration

Fasting plasma glucose was analyzed using the glucose-oxidase enzymatic test with a commercial kit (Spinreact SAU, Girona, Spain). The glucose levels were determined spectrophotometrically at 540 nm by using a microplate reader (Biotek HT Sinergy, Vermont, VT, USA). Plasma insulin concentration was quantified at 450 nm by using an ultrasensitive rat insulin enzyme immunoassay kit (Mercodia AB, Uppsala, Sweden) with a microplate reader (Biotek HT Sinergy). Plasma concentrations of both fasting glucose and insulin were used to calculate insulin resistance [homeostasis model assessment (HOMA)-IR] and insulin secretion [HOMA-β] indexes with the following formulas: HOMA-IR = fasting insulin (µU/mL) × fasting glucose (mM)/22.5; HOMA-β = 20 × fasting insulin (µU/mL)/[fasting glucose (mM) − 3.5] [19]. The Quantitative Insulin Sensitivity Check Index (QUICKI) was also calculated using the following equation. QUICKI = 1/log fasting insulin (µU/mL) + log fasting glucose (mg/dL) [20].

### 2.6. Pancreatic Islet Isolation and Determination of Insulin Secretion

The islets of Langerhans were isolated by collagenase digestion of the pancreas, according to Rafacho et al., 2007 [21]. The pancreas was transferred to a mortar, washed with Hanks solution (Sigma-Aldrich, St. Louis, MO, USA), and cut in small pieces with scissors. The islets were isolated by collagenase P digestion (Roche Diagnostics, Basel, Switzerland) with vigorous hand shaking for 3 min at 37 °C. The resulting suspension was washed 3 times with Hanks solution (Sigma-Aldrich) and islets were allowed to sediment for 4 min. One aliquot of the suspension was placed into a petri dish with Hanks solution supplemented with 1 mg/mL of 0.1% bovine serum albumin (BSA). Isolated islets were handpicked using a micropipette under a stereomicroscope (Nikon, Tokyo,‎ Japan). The isolated islets of each animal were transferred to the wells (4 islets/well) in 0.5 mL of Krebs-Ringer buffer solution supplemented with 0.5% of BSA, which contain either 0, 2, 6, 8, or 10 mM glucose. Every plate was incubated for 60 min at 37 °C with 95 % O_2_-5% CO_2_. At the end of the incubation, an aliquot of the medium was withdrawn to measure the insulin concentration as described above.

### 2.7. Pancreas Histopathological Analysis

Each isolated pancreas was fixed in buffered 10% formaldehyde, embedded in paraffin, cut in sections of 5 µm, and stained with conventional hematoxylin-eosin (HE). The organs were subsequently studied under a Zeiss Axioskop 2 microscope (Zeiss Microlmaging GmbH, Jena, Germany), which was equipped with the image analysis software package AxioVision 4.6 (Carl Zeiss Microlmaging GmbH, Jena, Germany) to calculate the morphometric parameters. The analysis was made in 2 to 4 slices per animal, which measured a minimum of 100 islets per treatment under a 40× objective. The perimeter was defined by manual drawing and the mean area of islets was expressed in µm^2^. The analyst was blind to the treatment received by the rat from which the sample under analysis was obtained. 

### 2.8. Statistical Analysis

Data are presented as the mean value ± SEM (8–10 animals per group). Differences between groups were analyzed using unpaired Student’s *t*-test or one or two-way analysis of variance (ANOVA), which was followed by post hoc Bonferroni multiple comparison test. GraphPad Prism 5 software (San Diego, CA, USA) was used and differences were considered significant at *p* < 0.05.

## 3. Results

### 3.1. Tactile Allodynia

**Figure 1:**
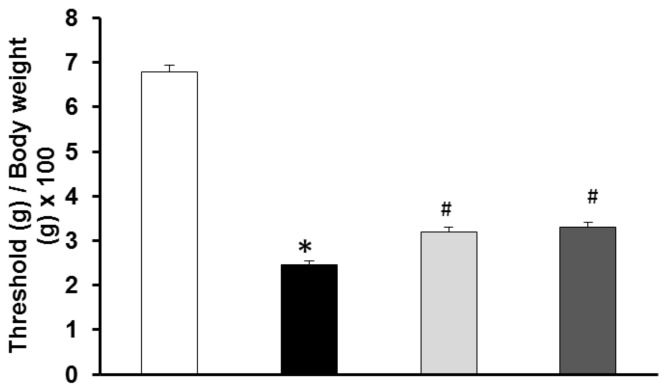
Mechanical sensitivity in 19-week-old rats. Experimental groups: control Zucker lean rats (ZLR, white bar), control Zucker fatty rats (ZFR, black bar), ZFR treated for 12 weeks (from week 8 to week 20) with 750 mg/kg/day of the egg white hydrolyzed with pepsin (light grey bar) and ZFR treated for 12 weeks with 750 mg/kg/day of the egg white hydrolyzed with aminopeptidase (dark grey bar). Bars show means ± SEM. One-way ANOVA was used. * *p <* 0.05 vs. ZLR, # *p <* 0.05 vs. ZFR (*n* ≥ 9 for all groups).

In order to avoid influence of the size of the animals, the threshold for mechanical sensitivity has been adjusted for body weight. Body weight of the different groups were ZLR: 404.0 ± 9.10 g, ZFR: 546.60 ± 14.04 g, ZFR + H1: 544 ± 8.17 g, and ZFR + H2: 538.10 ± 12.54 g. As shown in Figure 1, mechanical sensitivity was significantly reduced in 19–20 weeks old ZFR compared to the lean rats, which was a sign of tactile allodynia. The treatment with either HEW1 or HEW2 significantly increased the threshold of mechanical sensitivity in ZFR (*p* < 0.05) even though the levels did not reach the normal values of ZLR in either case.

### 3.2. Plasma Glucose, Plasma Insulin, and Indexes of Insulin Resistance

ZFR exhibited glucose levels similar to their lean age-matched controls and no significant differences were observed in the animals treated with either HEW1 or HEW2 (see Table 1). The insulin level was significantly higher in ZFR than in ZLR and the chronic treatment with HEW1 significantly decreased this value. Insulin was also decreased in ZFR treated with HEW2, but no significant differences were detected between this group of animals and ZFR (see Table 1).

Consequently, ZFR showed significantly higher values of the indexes of insulin resistance (HOMA-IR), pancreatic β cell functionality (HOMA-β), and a decreased insulin sensitivity index (QUICKI) (see Table 1) when compared to the lean rats. HEW1 treatment reduced HOMA-IR and HOMA-β and increased the value of QUICKI in comparison with non-treated ZFR. A similar tendency was observed in ZFR treated with HEW2, but no significant differences were reached in this case (see Table 1).

### 3.3. Insulin Secretion by Isolated Pancreas Islets

**Figure 2:**
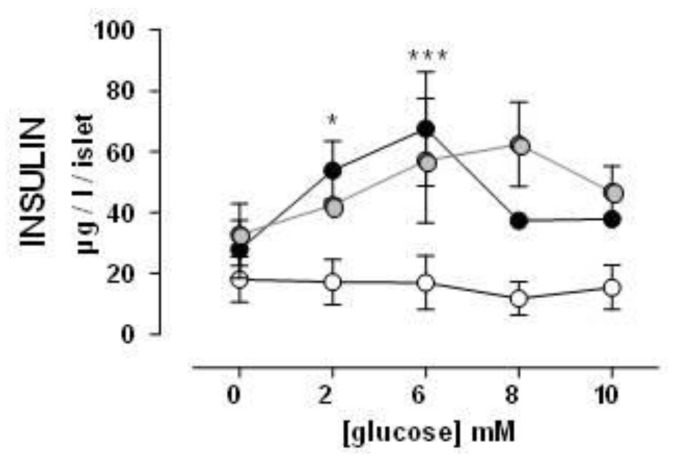
Insulin secretion by isolated pancreatic islets in response to the stimulus of different glucose concentrations. Experimental groups: Zucker lean rats (ZLR, white circles), control Zucker fatty rats (ZFR, black circles), ZFR treated for 12 weeks (from week 8 to week 20) with 750 mg/kg/day of the egg white hydrolyzed with pepsin (grey circles). Results are means ± SEM. Two-way ANOVA was used. * *p <* 0.05 and *** *p <* 0.001 vs ZLR (*n* ≥ 9 for all groups).

Glucose-stimulated insulin secretion by the islets of ZFR was increased compared to ZLR. Isolated islets of ZFR produced an increased insulin secretion, which was glucose dependent until the dosage of 6 mM. At the highest doses of glucose (8 and 10 mM), ZFR showed a noteworthy decrease in the insulin secretion. HEW1 treated ZFR and control ZFR showed similar responses, but in HEW1 group the noteworthy decrease on insulin secretion was observed only when pancreatic islets were stimulated with 10 mM of glucose (see Figure 2).

Since no significant differences were observed in either glucose or insulin levels in ZFR treated with HEW2 compared to ZFR, the insulin secretion by isolated pancreatic islets was not evaluated in this group.

### 3.4. Pancreas Histology

**Figure 3:**
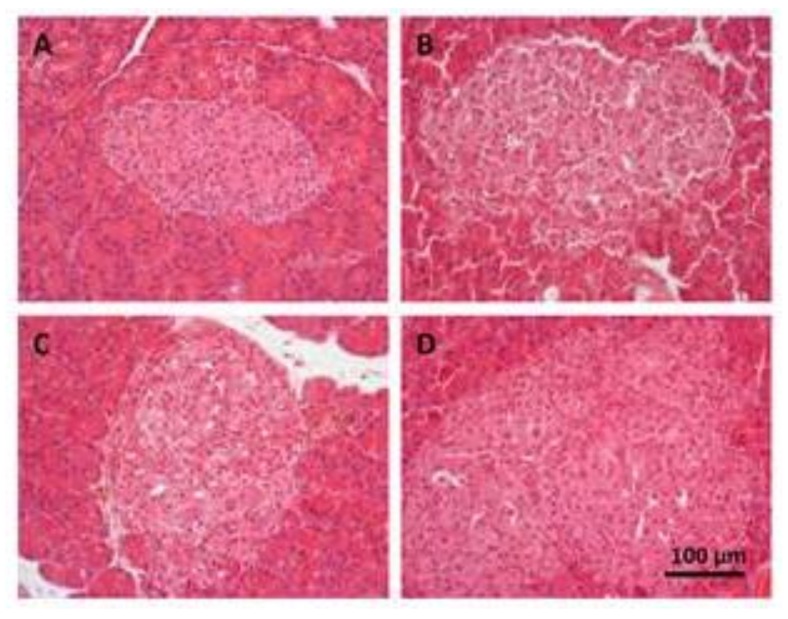
Optical microscope images (40×) of pancreatic sections stained with hematoxylin-eosin of 20-weeks old rats. (**A**) control Zucker lean rats (ZLR); (**B**) control Zucker fatty rats (ZFR); (**C**) ZFR treated for 12 weeks with 750 mg/kg/day of egg white hydrolyzed with pepsin; and (**D**) ZFR treated for 12 weeks with 750 mg/kg/day of egg white hydrolyzed with aminopeptidase. Bar: 100 µm.

Pancreas of ZLR showed no signs of pathology including no extravascular T or B-lymphocytes presented. Typically, rat pancreas tissue under the microscope consists of small and rounded aggregates of moderely eosinophilic cells including small peripheral cells and larger β-cells in the center (see Figure 3A).

**Figure 4:**
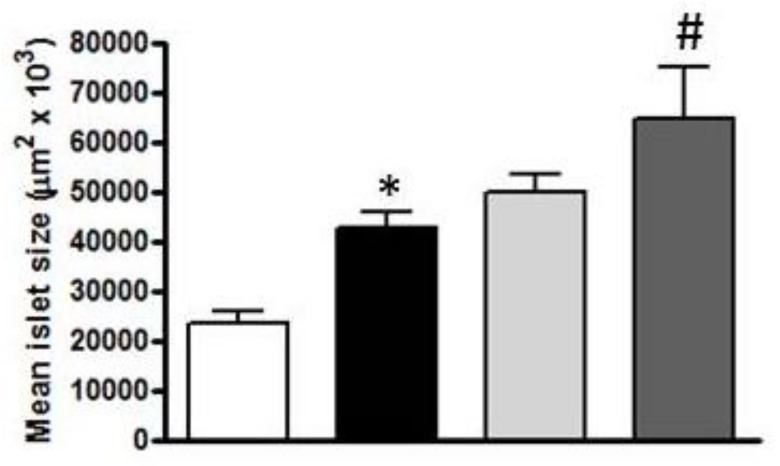
Mean islet size (µm^2^ × 10^3^) of rats at 20 weeks of age. The analysis was made in two to four slices per animal, which measured a minimum of 100 islets per treatment under a 40× objective. Experimental groups: control Zucker lean drank water (ZLR; white bar), control Zucker fatty rats (ZFR, black bar), ZFR treated for 12 weeks (from week eight to week 20) with 750 mg/kg/day of egg white hydrolyzed with pepsin (light grey bar) and ZFR treated for 12 weeks with 750 mg/kg/day of egg white hydrolyzed with aminopeptidase (dark grey bar). Bars show means ± SEM. Student *t*-test was used. * *p <* 0.05 vs. ZLR, # *p <* 0.05 vs. control ZFR (*n* ≥ 9 for all groups).

Mean islet size of ZLR was 23.5 × 10^3^ µm^2^ (see Figure 4). In contrast, all groups of obese ZFR rats showed dramatic changes in islet morphology (see Figure 3B–D). Most of the islets were enlarged, which shows hypertrophy/hyperplasia with irregular forms especially in the ZFR control group. Mean islet size was approximately two times larger than in ZLR, which ranged from 42.5 × 10^3^ µm^2^ in ZFR to 49.9 × 10^3^ µm^2^ in ZFR treated with HEW1 and 64.7 × 10^3^ µm^2^ in the group treated with HEW2 (see Figure 4).

## 4. Discussion

In this study, the ZFR was used as a genetic model of obesity and MS. These animals, at the end of the experimental period, showed similar glucose levels, but higher insulin levels than their age-matched lean controls. In addition, insulin secretory activity of islets was impaired and pancreas histology presented signs of pathology as well as exhibit tactile allodynia. These results agree with previous reports, which indicate that ZFR suffers metabolic abnormalities characteristic of prediabetes (hyperinsulinemia or impaired glucose tolerance in the absence of overt hyperglycemia), together with hypertriglyceridemia and/or increased non-esterified fatty acid abundance and hypercholesterolemia during their whole life span as well as end-organ damage associated with these conditions [22,23].

While no significant differences were found between ZFR and ZLR regarding fasting blood glucose and plasma insulin concentrations were higher in ZFR than in ZLR. Therefore, ZFR presented significantly higher values of the insulin resistance (HOMA-IR) and pancreatic β cells functionality (HOMA-β) indexes and a decreased insulin sensitivity index (QUICKI), which indicate the development of insulin resistance and β-pancreatic cell dysfunction. Unlike diabetes, the metabolic syndrome condition may happen without glucose elevation (at least at initial stages) but with modifications in plasma insulin and obesity. This occurs not only in rodents but also in humans. The results obtained are in agreement with previous findings, which report normal glucose levels and hyperinsulinemia in ZFR [5]. This develops hyperinsulinemia without hyperglycemia as early as the seventh week of age [24]. Interestingly, treatment with HEW1 significantly reduced plasma insulin concentration. HOMA-IR and HOMA-β it increased the QUICKI index in ZFR. Wang et al. [16] found that the administration of an egg lysozyme hydrolysate with alcalase did not change the plasma insulin levels of Zucker diabetic fatty rats (ZDF). However, there are several other studies that have demonstrated that dietary protein hydrolysates improve glucose metabolism in other experimental models of MS [25] as well as in human patients [26]. Bong et al. [25], observed a decrease in the HOMA-IR index after the administration of a corn protein hydrolysate to rats with diet-induced obesity and similar results were also found in ZFR consuming rice-derived phytochemicals [27]. Moreover, the increase in the QUICKI index observed in HEW1-treated ZFR suggests that these animals developed a compensatory mechanism for increasing insulin sensitivity. In patients with DM2, the administration of marine peptides increases the QUICKI index and the sensitivity to the effects of insulin [26].

In the insulin-resistant state, pancreatic islets usually respond by increasing insulin secretion to maintain normoglycaemia (β cell compensation). Therefore, hyperglycaemia, once established, promotes a further series of mechanisms that eventually cause severe β cell failure and DM2 [28,29]. This condition of insulin resistance could explain the results found in glucose-stimulated insulin secretion by the islets of animals. While no increased insulin secretion was observed in pancreatic islets of ZLR after glucose stimulation, ZFR showed a high insulin secretion until dose 6 mM of glucose. Higher doses of glucose in isolated islets, where no compensatory mechanisms can exist, caused β cell failure and the decrease of insulin secretion. Pancreatic islets of ZFR treated with HEW1 and control ZFR showed similar responses. However, in the HEW1 treated animals, the β-cell failure occurred only at a maximum dose of glucose (10 mM). These results also suggest a lower grade of insulin resistance for the HEW1 treated group than in ZFR, which agrees with the improvement observed in plasma on insulin levels and insulin resistance indexes. In fact, the histological study of the pancreas revealed significant changes in islets morphology, which shows hypertrophy, elongated cells, and irregular shapes. These factors are associated with functional damage to the β cells. Augstein, & Salzsieder [30] described substantial alterations in ZFR islets at 14 weeks of age: hyperplasia and/or hypertrophy with numerous islets considerably enlarged, β cell vacuolation, and hemorrhage into the islet tissue from dilated blood vessels. Hypertrophy of pancreatic cells was also observed in ZFR treated with both hydrolysates. Furthermore, HEW2 treatment aggravated hyperplasia/hypertrophy development in ZFR. More studies should be performed to explain these observations. In previous works we also observed different physiological responses between HEW1 and HEW2. The beneficial effect of HEW1 in fat accumulation, hepatic steatosis, and dyslipidemia on Zucker fatty rat was demonstrated. However, HEW2 did not show the same effects on the same experimental model [18]. In fact, the different outcomes of the administration of both hydrolysates produced with different enzymes, pepsin, or aminopeptidase, pointed at their differential peptide composition as responsible for the bioactivity.

In our study, ZFR, which have a bigger weight than ZLR ones, have a decreased mechanical sensitivity and tactile allodynia, which is a sign of peripheral neuropathy. It is in agreement with previous reports [22], which describe the presence of allodynia [22], thermal hyperalgesia [31] or mechanical hyperalgesia [32] in this model of prediabetes condition. Similar results have also been found in obese patients who develop polyneuropathy even in presence of normoglycemia and in prediabetes [33]. This phenomenon is also found in animal models DM1 and DM2 when hyperinsulinemia and hyperglycemia are well established [34,35]. The etiology of the development of neuropathy prior to hyperglycemia is not well understood, but it could implicate obesity, impaired fasting glycaemia/glucose tolerance, elevated triglycerides, cholesterol, and non-esterified fatty acids as well as oxidative–nitrative stress [22]. Treatment with HEW1 and HEW2 reduced tactile allodynia, which could be attributed to the antioxidant and hypolipidemic properties observed in vitro in both egg white hydrolysates [17]. Products with lipid-lowering and antioxidant properties have been reported to ameliorate the peripheral nerve function in ZFR and ZDF [22,36] and certain proteins hydrolysates that have been evaluated in peripheral neuropathy were shown to improve this condition [37].

## 5. Conclusions

In summary, these results suggest an improvement in glucose homeostasis in ZFR after the administration of an egg white hydrolysate with pepsin (HEW1). A key feature of metabolic syndrome is insulin resistance. In order to gain further insight into the mechanisms and pathways implicated in the protective effects of HEW1 in this context, more research is needed with appropriated experimental models of diabetes including gene expression analyses in different metabolic tissues. Moreover, we are aware that before using HEW as a functional food ingredient, it would be necessary to carry out clinical studies to demonstrate their efficiency in humans.

## 6. Patents

Patent number (PCT/ES2014/070880), 28 November 2014.

## Figures and Tables

**Table 1:** Plasma glucose, plasma insulin and indexes of insulin resistance and insulin sensitivity.

	ZLR	ZFR	ZFR + HEW1	ZFR + HEW2
Glucose (mg/dL)	76.6 ± 3.4	85.9 ± 2.6	89.2 ± 1.7	93.2 ± 2.0
Insulin (ng/mL)	6.43 ± 1.0	25.7 ± 4.8 *	14.3 ± 2.1 #	18.4 ± 3.1
HOMA-IR	24.5 ± 2.5	139.8 ± 20.5 *	79.5 ± 11.5 #	106.4 ± 17.4
HOMA-β	146.1 ± 39.2	415.7 ± 65.7 *	199.6 ± 35.7 #	234.7 ± 42.8
QUICKI	0.37 ± 0.006	0.29 ± 0.007 *	0.31 ± 0.007 #	0.30 ± 0.006

Fasting glycemia, plasma insulin concentration, insulin resistance index (HOMA-IR), insulin secretion index (HOMA-β), and quantitative index of insulin sensitivity (QUICKI) measured in 20-week-old rats. Experimental groups: control Zucker lean rats (ZLR), control Zucker fatty rats (ZFR), ZFR treated for 12 weeks with 750 mg/kg/day of egg white hydrolyzed with pepsin (ZFR + HEW1), and ZFR treated for 12 weeks with 750 mg/kg/day of egg white hydrolyzed with aminopeptidase (ZFR + HEW2). Data are the mean ± SEM. The student *t*-test was used. * *p <* 0.05 vs. ZLR, # *p* < 0.05 vs. control ZFR (*n* ≥ 9 for all groups).
